# Melatonin in the Prophylaxis of SARS-CoV-2 Infection in Healthcare Workers (MeCOVID): A Randomised Clinical Trial

**DOI:** 10.3390/jcm11041139

**Published:** 2022-02-21

**Authors:** Irene García-García, Enrique Seco-Meseguer, Pilar Ruiz-Seco, Gema Navarro-Jimenez, Raúl Martínez-Porqueras, María Espinosa-Díaz, Juan José Ortega-Albás, Iñigo Sagastagoitia, María Teresa García-Morales, María Jiménez-González, Lucía Martínez de Soto, Ana Isabel Bajo-Martínez, María del Palacio-Tamarit, Raquel López-García, Lucía Díaz-García, Javier Queiruga-Parada, Christine Giesen, Ana Pérez-Villena, Marta de Castro-Martínez, Juan J. González-García, Miguel Rodriguez-Rubio, Pedro de la Oliva, José R. Arribas, Antonio J. Carcas, Alberto M. Borobia

**Affiliations:** 1 Clinical Pharmacology Department, La Paz University Hospital-IdiPAZ, 28046 Madrid, Spain; irene.ucicec@gmail.com (I.G.-G.); enriquesm.ucicec@gmail.com (E.S.-M.); jimenezglezmaria@gmail.com (M.J.-G.); luciamds.ucicec@gmail.com (L.M.d.S.); luciadiaz.ucicec@gmail.com (L.D.-G.); javier.ucicec@gmail.com (J.Q.-P.); 2 Spanish Clinical Research Network (SCReN), 28046 Madrid, Spain; mariateresa.garcia@h12o.es; 3 Internal Medicine Department, Infanta Sofía University Hospital, 28702 San Sebastián de los Reyes, Spain; mprseco@salud.madrid.org (P.R.-S.); gema.nvj@gmail.com (G.N.-J.); 4 Internal Medicine Department, Hospital Universitario 12 de Octubre, 28041 Madrid, Spain; raul.martinezporqueras@gmail.com (R.M.-P.); maclamana@gmail.com (M.E.-D.); mariadelpalaciotamarit@yahoo.es (M.d.P.-T.); martadecastro84@gmail.com (M.d.C.-M.); 5 Sleep Unit, Hospital General Universitario de Castellón, 12004 Castellón de la Plana, Spain; jjoralbas@gmail.com (J.J.O.-A.); raquel_garnet@hotmail.com (R.L.-G.); 6 Internal Medicine/Infectious Diseases Department, Hospital Clínico San Carlos, IdiSSC, 28040 Madrid, Spain; sagastita@hotmail.com; 7 Instituto de Investigación Sanitaria Hospital 12 de Octubre (imas12), 28041 Madrid, Spain; 8 Infectious Diseases Unit, La Paz University Hospital-IdiPAZ, 28046 Madrid, Spain; juangonzalezgar@gmail.com (J.J.G.-G.); joser.arribas@salud.madrid.org (J.R.A.); 9 Emergency Department, Infanta Sofía University Hospital, 28702 San Sebastián de los Reyes, Spain; aisabel.bajom@salud.madrid.org; 10 Preventive Medicine Unit, Infanta Sofia University Hospital, 28702 San Sebastián de los Reyes, Spain; christine.giesen@salud.madrid.org; 11 Pediatric Department, Infanta Sofia University Hospital, 28702 San Sebastián de los Reyes, Spain; aperezv@salud.madrid.org; 12 School of Medicine, Universidad Autónoma de Madrid, 28029 Madrid, Spain; rodriguezrubio.miguel@gmail.com (M.R.-R.); pedro.oliva@salud.madrid.org (P.d.l.O.); 13 Centro de Investigación Biomédica en Red de Enfermedades Infecciosas (CIBERINFEC), 28029 Madrid, Spain; 14 Pediatric Intensive Care Department, La Paz University Hospital-IdiPAZ, Paseo de la Castellana, 261, 28046 Madrid, Spain

**Keywords:** SARS-CoV-2, melatonin, prophylaxis, COVID-19, healthcare workers

## Abstract

We evaluated in this randomised, double-blind clinical trial the efficacy of melatonin as a prophylactic treatment for prevention of SARS-CoV-2 infection among healthcare workers at high risk of SARS-CoV-2 exposure. Healthcare workers fulfilling inclusion criteria were recruited in five hospitals in Spain and were randomised 1:1 to receive melatonin 2 mg administered orally for 12 weeks or placebo. The main outcome was the number of SARS-CoV-2 infections. A total of 344 volunteers were screened, and 314 were randomised: 151 to placebo and 163 to melatonin; 308 received the study treatment (148 placebo; 160 melatonin). We detected 13 SARS-CoV-2 infections, 2.6% in the placebo arm and 5.5% in the melatonin arm (*p* = 0.200). A total of 294 adverse events were detected in 127 participants (139 in placebo; 155 in melatonin). We found a statistically significant difference in the incidence of adverse events related to treatment: 43 in the placebo arm and 67 in the melatonin arm (*p* = 0.040), and in the number of participants suffering from somnolence related to treatment: 8.8% (*n* = 14) in the melatonin versus 1.4% (*n* = 2) in the placebo arm (*p* = 0.008). No severe adverse events related to treatment were reported. We cannot confirm our hypothesis that administration of melatonin prevents the development of SARS-CoV-2 infection in healthcare workers.

## 1. Introduction

In December 2019, a novel coronavirus identified as SARS-CoV-2 (Severe Acute Respiratory Syndrome-coronavirus) began to spread in Wuhan, China. The World Health Organization (WHO) announced this new virus as being responsible for the outbreak of coronavirus disease 2019 (COVID-19) in February 2020 [1]. COVID-19 has been divided into five clinical types: asymptomatic, mild, moderate, severe, and critical cases. The main reported symptoms include fever or chills, cough, shortness of breath or difficulty breathing, fatigue, muscle or body aches, headache and loss of taste or smell. Although 80% of the cases are mild, the infection can develop into pneumonia, [2]. Infected males (as twice as likely as females) and patients aged 15–29 years, healthcare workers and nursing home residents, and those with diabetes, chronic pulmonary disease, cardiovascular disease, hypertension, chronic kidney disease, dementia, severe obesity, or cancer are more prone to suffer from coronavirus infection [3].

The COVID-19 pandemic continues to constitute a public health emergency of international concern associated with devastating effects on populations, social structures, and economic growth [4,5]. The global research community has faced urgent calls for the development of rapid diagnostic tools, effective treatment protocols, and most importantly, prophylaxis against the pathogen, such as vaccines.

Melatonin (N-acetyl-methoxy-tryptamine) is a hormone primarily released by the pineal gland. Apart from the brain, melatonin is also synthesized in lymphocytes, bone marrow, the eyes and gastrointestinal tract [6]. The endogenous rhythm of secretion is generated by the suprachiasmatic nuclei and entrained to the light/dark cycle. The primary physiological function of melatonin, whose secretion adjusts to night length, is to act as an endogenous synchronizer to convey information concerning the daily cycle in order to stabilize circadian rhythms, to reinforce them and to maintain their mutual phase-relationship [7]. Melatonin is also known to have anti-inflammatory, anti-oxidant and immune-enhancing features [8,9,10]. Given these positive effects, it was long ago suggested for the treatment of SARS-CoV-1 [11] and Ebola [12], and it has been proven beneficial in other viral infections [13]. Its administration has also been considered in patients with SARS-CoV-2 infection during the pandemic [14,15].

Furthermore, melatonin could have prophylactic properties against COVID-19; SARS-CoV-2 infection has a predilection for patients that exhibit lower melatonin production as a common factor: older age, males and patients in whom melatonin is somehow involved, such as endocrine, metabolic, or cardiovascular diseases [16,17,18,19]. Melatonin blood levels are higher in children; the disease in this population group is less frequent than in adults, and most cases are benign or moderate [20]. This, along with the known features of melatonin, led us to consider the benefits of using this molecule not only as an adjuvant treatment for SARS-CoV-2, but also preventively. In consequence, we designed a clinical trial aiming to evaluate the efficacy of melatonin as a prophylactic treatment for the prevention of symptomatic SARS-CoV-2 infection among healthcare workers (HCWs) at high risk of SARS-CoV-2 exposure.

## 2. Materials and Methods

### 2.1. Trial Design

Between April and December 2020, we conducted a multicentre, randomised, parallel, 2-arm, double-blind and placebo-controlled clinical trial, to evaluate the efficacy of melatonin versus placebo in the prophylaxis of SARS-CoV-2 infections among healthcare workers. The lead institution was La Paz University Hospital (Madrid, Spain), and the other participating sites were Infanta Sofía, Clínico San Carlos and 12 de Octubre Hospitals (Madrid) and Castellón Hospital (Comunidad Valenciana, Spain). The study was approved by the Ethics Committee of La Paz University Hospital and by the Spanish Agency of Medicines and Health Products (EudraCT: 2020-001530-35; NCT04353128). The trial was undertaken in accordance with the Good Clinical Practice guidelines and the Declaration of Helsinki. All participants provided written informed consent.

### 2.2. Study Population

We recruited healthcare workers at high risk of SARS-CoV-2 exposure. The main selection criteria were not having a previous COVID-19 diagnosis and having a negative serologic rapid test (IgM/IgG) result before randomisation. The detailed inclusion and exclusion criteria have been previously described [21].

### 2.3. Randomisation, Treatment and Blinding

After inclusion in the trial, the participants were randomly assigned in a 1:1 ratio to either of 2 arms: Melatonin (Circadin^®^, Exeltis Healthcare, Alcobendas, Spain) 2 mg orally before bedtime for 12 weeks or identical-looking placebo (Laboratorios Liconsa, Azuqueca de Henares, Spain) orally before bedtime for 12 weeks (comparator). The randomisation sequence was created using SAS version 9.4 statistical software (procedure ‘PROC PLAN’) with a 1:1 allocation. No randomisation seed was specified. The randomisation seed was generated taking the hour of the computer where the program was executed. Randomisation was performed centrally through the electronic system RedCAP^®^ in order to conceal the sequence until interventions were assigned. Participants, caregivers, and those assessing the outcomes were blinded to group assignment.

### 2.4. Study Procedures

Healthcare workers from the participating sites were invited to participate in the study. Those volunteers who met all of the inclusion and none of the exclusion criteria were selected. Before undergoing any study procedure, we confirmed that the participants had signed the informed consent. The volunteers who agreed to participate in this clinical trial had to perform a total of four face-to-face visits: Basal or screening (D1), Week 4 (D30), Week 8 (W8) and Week 12 (W12). On the screening day, after signing informed consent, medical history was recorded, a targeted physical examination was performed, and vital signs were measured. Additionally, serologic rapid test (IgM/IgG) was performed. Only volunteers having a negative serologic rapid test were included. Participants meeting all of the inclusion criteria and none of the exclusion criteria were randomised, and the assigned treatment was dispensed. On the following visits (D30, W8 and W12 (± 5 days)), medical history (anamnesis of any clinical changes or new medication) and vital signs were recorded, physical examination was performed if deemed necessary by the physician, adverse events were recorded, and a serologic rapid test (IgM/IgG) was performed. A follow-up phone visit was performed 4 weeks after the last intake of the study drug to collect information about adverse events and medical history. During the study, from the start of study treatment until week 12, participants were expected to daily enter in an online application data concerning treatment administration, adverse events, food intake, exercise, sleeping hours, risk of exposure to SARS-CoV-2 and other data related to the effects of melatonin. The detailed procedures have been previously published [21].

### 2.5. Outcome Variables

The study’s primary endpoint was the number of SARS-CoV-2 symptomatic infections confirmed by polymerase chain reaction (PCR) test or serologic test or according to each centre diagnosis protocol. This variable was measured until the end of treatment for each participant (until the date of the last dose taken by each participant).

Due to the low incidence of SARS-CoV-2 infections throughout the study, we finally decided to analyse as primary endpoint the number of all SARS-CoV-2 infections (symptomatic and asymptomatic) confirmed by polymerase chain reaction (PCR) test or serologic test or according to each centre diagnosis protocol. Secondary endpoints as per protocol have been previously published [21]. Due to the low incidence of SARS-CoV-2 infections during the study, most of the secondary endpoints could not be analysed.

### 2.6. Statistical Analysis

We calculated the sample size to detect an absolute difference of 20% in the number of infections, assuming a 54% rate of infection in healthcare workers in the placebo arm. For a statistical power of at least 90% and an alpha error of 5%, the number of participants needed was 225 per arm.

There were 3 study populations: the intention-to-treat (ITT) population, defined as all randomised participants who met the selection criteria; the per protocol (PP) population, defined as randomised participants who met the inclusion criteria, took the study medication and completed the study; and the safety population, defined as all randomised participants who took at least one treatment dose. The main study analysis was performed using the ITT population.

The normality of the variables was studied with the Shapiro–Wilks test. For the comparison between study treatment groups, Pearson chi-square test (or Fisher exact test, if necessary) was performed for qualitative variables, and Mann–Whitney U test and Kruskal–Wallis test were used in the case of quantitative variables. The infection probability functions were adjusted with the Kaplan–Meier method. For the study of the influence of treatment on the risk of infection, the hazard ratio and 95% confidence intervals were calculated with a Cox regression adjusted for sex, age and SARS-CoV-2 risk factors. Statistical analysis was performed using R v4.1.1 software.

### 2.7. Role of the Funding Source

This clinical trial was not funded. The sponsor is the Investigator Coordinator (Dr. Alberto M. Borobia. La Paz University Hospital, Madrid, Spain). Circadin^®^ and placebo were provided by Exeltis Healthcare, S.L. Serologic rapid test (IgM/IgG) was provided by the Spanish Ministry of Health. The clinical trial was designed and the data analysed by the senior authors and the biostatistician.

## 3. Results

### 3.1. Population

**Figure 1:**
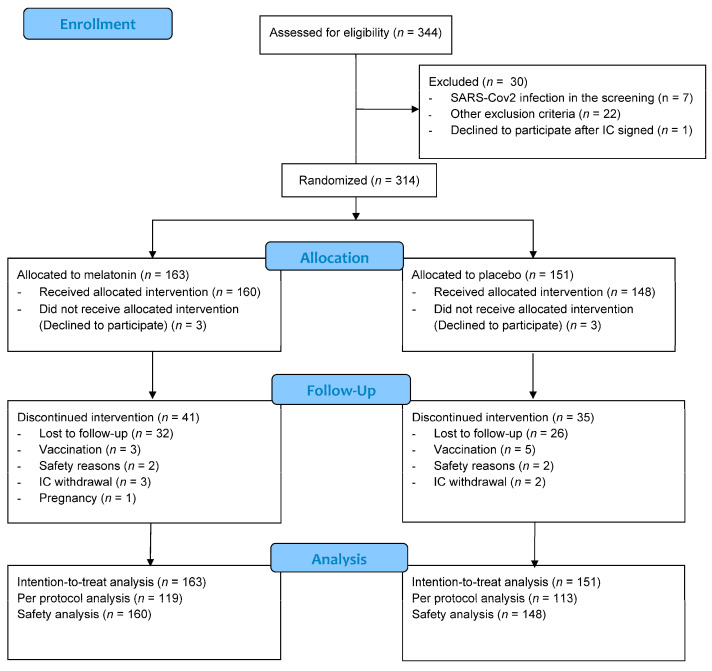
Flowchart of participants included. IC: Informed consent.

A total of 344 participants underwent the screening visit for the study and signed the informed consent; however, 30 of them were excluded due to different reasons. The remaining 314 participants were randomised, 163 to melatonin and 151 to placebo constituting the ITT population (Figure 1). The median age in the ITT population was 40 years [IQR 32, 49], and women represented 81.2% (*n* = 255) of the participants in this population. A total of 120 participants had at least one comorbidity: 42.4% (*n* = 64) of the participants receiving placebo and 34.4% (*n* = 56) of the participants receiving melatonin. Forty participants had at least one risk factor for severe COVID-19 (12.7%): 24 in the placebo arm and 16 in the melatonin arm (Table 1). Other demographic factors were compared between groups (Table 1).

### 3.2. Primary Outcome

The primary endpoint was the number of SARS-CoV-2 infections (symptomatic and asymptomatic) confirmed by PCR test or serologic test or according to each centre diagnosis protocol.

We detected 13 SARS-CoV-2 infections during the follow-up, 4 (2.6%) in the placebo arm and 9 (5.5%) in the melatonin arm (most of them asymptomatic or paucisymtomatic, two symptomatic and no cases of moderate/severe COVID-19). The analysis of the primary efficacy variable in the ITT and PP population showed no statistically significant difference between treatment groups in the number of SARS-CoV-2 (COVID-19) infections (Table 2).

### 3.3. Secondary Outcomes

None of the participants infected was hospitalised. The number of infections stratified by risk of exposure to SARS-CoV-2 (high or low) was one of our secondary outcomes; however, we could not assess this endpoint due to insufficient data regarding the risk of exposure. Among the 13 participants that tested positive, data concerning risk of exposure were only available for four, and only one of these four had high risk of exposure. Nevertheless, all of the participants were healthcare workers that probably had a similar risk of exposure.

**Figure 2:**
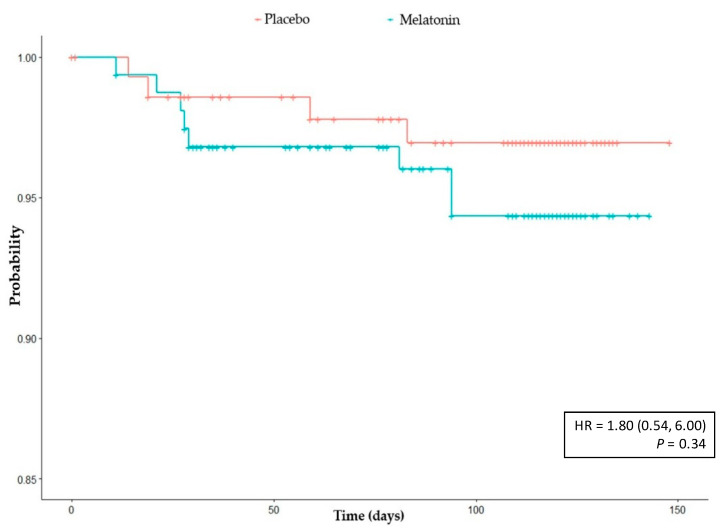
Kaplan–Meier analysis: time to infection. ITT population. Cox regression for the study of the influence of treatment was adjusted for sex, age and presence or absence of risk factors for Sars-CoV-2.

We also analysed the time from the start of treatment to the positive PCR test or serologic test; there was not a statistically significant difference between treatment arms in the time-to-event in the IIT (Figure 2) or in the PP population *p* = 0.12 [HR: 2.84 (0.54,6.00)].

Other variables of interest were percentage of males and females with a positive PCR or serologic test during the study and the percentage of males and females suffering from severe COVID-19. Among infected participants, 84.6% were female (*n* = 11). There was no statistically significant difference between positively tested males (3.4%) and females (4.3%) (*p* = 1). None of the infected participants suffered from severe COVID-19.

### 3.4. Safety Outcomes

A total of 294 adverse events (AEs) were detected throughout the study in 127 participants (139 in the placebo arm and 155 in the melatonin arm), most of them were graded as mild (78.34%), and none were graded as severe. A total of 110 AEs were related to the study treatment: 43 in the placebo arm (30.9% of the AEs reported for placebo) and 67 in melatonin (43.2% of the AEs reported for melatonin); the incidence of AEs related to treatment differed significantly between the two groups (*p* = 0.04).

Among AEs related to treatment, we found a statistically significant difference between groups in the number of participants suffering from somnolence: 8.8% (*n* = 14) of the participants that received melatonin versus 1.4% (*n* = 2) of the participants that received placebo (*p* = 0.008). The related AE most frequently detected was headache (*n* = 43); 15.6% of the participants receiving melatonin reported this AE, versus 12.2% of the participants receiving placebo. The second most frequent related AE was insomnia (*n* = 26), suffered by 9.4% of the participants in the placebo arm and 7.4% of the participants in the melatonin arm. Other AEs reported by at least three participants were: abnormal dreams, dizziness and dysmenorrhea. No statistically significant differences were found between treatment groups in these related AEs (Table 3). No severe adverse events related to treatment were reported.

## 4. Discussion

To date, COVID-19 has caused an unprecedented public health crisis and continues to represent a challenging situation for governments and healthcare systems. Over 304 million cases have been confirmed worldwide, and cumulative deaths have exceeded 5.4 million [22]. When the study was performed, there were no specific pharmacological treatments nor approved vaccines against COVID-19 infection in any part of the world. Hence, the search for effective prophylactic agents to tackle COVID-19, until vaccines were developed, was vital and urgent.

Some therapies have been considered as possible prophylaxis for SARS-CoV-2 infection. Hydroxychloroquine sulphate is a chloroquine analogue, commonly used for malaria, rheumatoid arthritis and systemic lupus erythematosus [23]. It possesses anti-inflammatory and immunomodulatory properties, including inhibition of cytokine (IL-1 and IL-6) production, inhibition of phospholipase A2 and matrix metalloproteinases, and modulation of B and T cell function [24]. Clinical trials were performed evaluating the use of hydroxychloroquine as pre-exposure prophylaxis in healthcare workers [25,26]. As a result, no clinical benefit was shown in significantly reducing COVID-19 incidence [23,25,26,27]. Tenofovir (TDF) and emtricitabine (FTC) are both reverse-transcriptase inhibitors with activity against human immunodeficiency virus (HIV) and hepatitis B virus, resulting in blockage of viral replication [28]. Observational studies found that patients receiving TDF/FTC had milder symptoms and a lower risk of hospitalisation [29,30]. Three randomised, controlled trials from Spain (EPICOS) [31], Argentina (CoviPrep) [32] and Colombia [33] are investigating the role of a tenofovir and emtricitabine combination for preexposure prophylaxis in healthcare workers within 8 to 12 weeks [28]. Nevertheless, no results have been published yet. The use of other antiviral drugs such as remdesivir might be effective, as it completely protected exposed macaques from MERS-CoV-induced clinical disease [34]. However, the prophylactic use of remdesivir is limited by its poor oral bioavailability and the lack of an oral formulation; moreover, drug pricing prevents its widespread access [35]. Virostatic-acting drugs such as neuraminidase inhibitors may prevent influenza virus infections and can also be used as post-exposure prophylaxis [35]. On the other hand, antiparasitic ivermectin’s ability to inhibit SARS-CoV-2 replication, which likely leads to lower infection rates, suggests it as a possible protective medication [36]. According to Cruciani M et al., there is limited evidence for the benefit of ivermectin for COVID-19 treatment and prophylaxis, and most of this evidence is of low quality [37]. In addition, other strategies, such as supplementation with vitamin D, may have an effect in restraining COVID-19 infection. The active form of vitamin D can induce the expression of angiotensin-converting enzyme 2 (ACE2) and regulate the immune system through different distinct mechanisms [38]. Despite its suitable safety profile and its broad accessibility, no clinical evidence is available yet [39].

Current literature suggests melatonin as a possible immunomodulator for SARS-CoV-2 infection; however, this article presents data from the first published clinical trial evaluating the efficacy of melatonin in the prophylaxis of SARS-CoV-2 infection, showing no significant difference compared to placebo. Regarding safety outcomes, the study showed an adequate safety profile for melatonin. No serious adverse events related to treatment were reported in either the melatonin or placebo group. The only related AE in which statistically significant differences were found between groups was somnolence, reported more frequently in those participants receiving melatonin. This effect was not unexpected and may be related to the mechanism of action of the drug itself, as it is commonly used to treat insomnia and other sleep disorders.

Some limitations need to be considered when interpreting these results. The needed sample size was not reached due to the start of clinical trials for COVID-19 vaccines in September 2020 in our country. Furthermore, the low incidence of SARS-CoV-2 infection during the investigation period led to a scarce number of events in the main study outcome: the number of positive cases in both placebo and treatment group was lower than expected when the study protocol was developed; therefore, although initial objectives of the study were intended to analyse only symptomatic cases, due to this low incidence, all SARS-CoV-2 infections were assessed. Despite these aspects, this was a properly designed randomised clinical trial, controlled with identical-looking placebo in which some interesting data were collected, leading to the first published clinical trial evaluating melatonin as prophylaxis for SARS-CoV-2 infection.

## 5. Conclusions

Despite the study’s limitations, the results obtained suggest a lack of efficacy of melatonin in preventing SARS-CoV-2 infection in healthcare workers at high risk of exposure.

## Figures and Tables

**Table 1:** Baseline data.

Parameter	Placebo	Melatonin
ITT Population (*N* = 314)	*N* = 151	*N* = 163
Age, years (median, [IQR])	39 [31, 49]	41 [32, 49.5]
Female sex, *n* (%)	123 (81.5%)	132 (81%)
Blood pressure (median, [IQR])		
Systolic	118.00 [110.00, 128.00]	116.00 [105.00, 125.00]
Diastolic	73.00 [66.00, 79.00]	73.00 [67.50, 78.00]
Heart rate (median, [IQR])	77.00 [68.00, 83.00]	75.00 [66.50, 83.00]
Weight (median, [IQR])	64.40 [57.05, 74.15]	63.60 [56.10, 71.50]
Height (median, [IQR])	1.65 [1.60, 1.70]	1.64 [1.58, 1.70]
Comorbidities ^a^ *n* (%)	64 (42.4)	56 (34.4)
Risk factor for severe COVID-19 ^b^	24 (15.9)	16 (9.8)
Hypertension ^c^	6 (4.0)	4 (2.5)
Dyslipidemia ^c^	4 (2.6)	3 (1.8)
Neoplasia ^c^	6 (4.0)	2 (1.2)
Respiratory diseases ^c^	8 (5.3)	7 (4.3)
Obesity ^c^	0 (0.0)	1 (0.6)
Heart diseases ^c^	1 (0.6)	0 (0.0)

^a^ Number of participants presenting at least one comorbidity. ^b^ Number of participants presenting at least one risk factor for severe COVID-19. ^c^ Number of participants presenting each of the risk factors for severe COVID-19.

**Table 2:** Main efficacy outcome.

SARS-CoV-2 Infections	Placebo *n* (%)	Melatonin *n* (%)	*p*-Value ^a^	RR (IC_95%_)	*p*-Value ^b^
ITT (*n* = 314)	4 (2.6)	9 (5.5)	0.20	2.02 (0.64, 6.45)	0.26
PP (*n* = 232)	4 (3.5)	9 (7.6)	0.18	2.05 (0.65, 6.50)	0.26

^a.^ Pearson chi-square test *p*-value for the main efficacy outcome (number of SARS-CoV-2 infections between groups). ^b.^ Relative Risk (RR) *p*-value.

**Table 3:** Safety outcomes.

Reported AEs	Placebo *N*^a^ = 148	Melatonin *N*^a^ =160	*p*
	*N*^b^ (%)	*N*^b^ (%)	
Related	31 (20.9)	44 (27.5)	1.000
Not Related	44 (29.7)	38 (23.8)	0.290
Related AEs *			
Abnormal dreams	4 (2.7)	3 (1.9)	0.917
Dizziness	1 (0.7)	2 (1.2)	1.000
Dysmenorrhoea	0 (0.0)	3 (1.9)	0.274
Headache	18 (12.2)	25 (15.6)	0.477
Insomnia	11 (7.4)	15 (9.4)	0.684
Somnolence	2 (1.4)	14 (8.8)	0.008 *

*N*^a^ = number of participants in the specified group. This is the Safety Population. This value is the denominator for the percentage calculations. *N*^b^ = Number of participants reporting at least one occurrence of the specified event category. * Related AEs reported by at least three participants.

## Data Availability

The data presented in this study are available on request from the corresponding author (Alberto M. Borobia; alberto.borobia@salud.madrid.org). The data are not publicly available because this was a sub-analysis of a cohort of patients containing information that could compromise the privacy of research participants.
